# Incidence, causes and risk factors for 30-day readmission after radical gastrectomy for gastric cancer: a retrospective study of 2,023 patients

**DOI:** 10.1038/s41598-018-28850-8

**Published:** 2018-07-12

**Authors:** Hua Xiao, Hu Quan, Shuguang Pan, Bin Yin, Wei Luo, Ming Tang, Yongzhong Ouyang, Wei Tang

**Affiliations:** Department of Gastroduodenal and Pancreatic Surgery, Hunan Cancer Hospital and the Affiliated Cancer Hospital of Xiangya School of Medicine, Central South University, 410013 Changsha, China

## Abstract

The aim of this retrospective study was to investigate the incidence of, causes and risk factors for readmission to hospital ≤30 days after discharge of patients who underwent radical gastrectomy for gastric cancer. A total of 2,023 patients underwent radical gastrectomy operations from November 2010 to July 2017 in our hospital. Of these, 60 patients (3.0%) were readmitted within 30 days after their original discharge. The median time span between the index discharge and readmission was 14 days and the median time for readmission was 8 days. The main reasons for readmission were intestinal obstruction (n = 10, 16.7%), intra-abdominal fluid collection (n = 9, 15.0%), abdominal pain (n = 7, 11.7%), nutritional difficulty (n = 4, 6.7%) and anastomotic leakage (n = 4, 6.7%). Five patients (8.3%) required intensive care and 4 patients (6.7%) died from sudden cardiac arrest, gastrointestinal bleeding, sepsis or multiple organ dysfunctions. Multivariate analysis revealed that post-operative complications (Odds Ratio = 5.116, 95% confidence interval: 2.885–9.073, P < 0.001) was the only independent risk factor for readmission. Thus, appropriate strategies on discharge and close follow-ups for these high-risk patients should be drawn up in order to enhance significantly their quality of care.

## Introduction

The early post-discharge period is a critical stage when patients may develop complications as a result of surgery, some requiring readmission to hospital. In fact, about 1 in 7 patients who were admitted to hospital for a major surgical procedure exhibited preventable adverse effects, which subsequently required readmission after ≤30 days^1^. Readmission following inpatient hospitalization is common and costly. In addition, several studies have indicated that readmission is associated with increased mortality and morbidity, a decreased quality of life and a significantly shorter 5-year overall survival time for cancer patients^2–4^. Thus, it has been considered as a measure of quality for surgical care and decreasing the proportion of readmissions is accepted as a clinical and healthcare policy priority^1,5^. The fourth most common cancer worldwide is gastric cancer, which is responsible for the second highest number of cancer-related deaths in China^6,7^. Gastrectomy with lymph node dissections for gastric cancer is the only therapeutic modality for cure, with morbidity and readmission rates ranged from 12.6% to 22.4%, and 2.2% to 15.4%, respectively^2,8–12^. The incidence and predictors for readmission after various types of surgery have been reported, based on a large database published by the American College of Surgeons National Surgical Quality Improvement Program. Unfortunately, this study did not include patients who underwent gastrectomy^13^. Identifying risk factors for readmission for specific patient populations and developing more targeted interventions is a prerequisite for reducing the incidence of readmission. The current study from a high-volume center in China was initiated to characterize the incidence, timing of, and reasons for readmission and to investigate the potential risk factors associated with readmission in patients who had underwent radical gastrectomy for gastric cancer.

## Materials and Methods

### Patients

A cohort of patients who underwent surgery for gastric cancer between November 2010 and July 2017 were identified in the database of the Affiliated Cancer Hospital of Xiangya School of Medicine, China. Eligibility criteria for this study were: patients undergoing radical gastrectomy for primary gastric cancer; ≥18 years of age; had adequate organ functions. Exclusion criteria were: patients who required emergency surgery; underwent an operation with palliative intent; had other synchronous malignancies; had remnants of gastric cancer; died during the initial hospitalization; or with incomplete clinical data. Of 2,482 patients with gastric cancer who underwent surgery in this period, 459 were excluded because of non-resection surgery (n = 136), palliative surgery (n = 127), remnants of gastric cancer (n = 25), other synchronous malignancies (n = 21), emergency surgery (n = 12), died during the initial hospitalization (n = 5) or had incomplete clinical data (n = 133). Therefore in total, data from 2,023 patients were analyzed in this retrospective study.

### Surgical procedures and peri-operative management

Surgeons with sufficient experience of radical gastrectomy performed all operations. Each tumor was pathologically diagnosed and staged according to the 7th UICC (Union for International Cancer Control) TNM (Tumor-Lymph Node-Metastasis) Staging System of Gastric Cancer^14^. Lymph node dissection and gastric reconstruction were determined according to the Japanese gastric cancer treatment guidelines^15^. Patients with clinical T1a or T1b with N0 underwent D1 or D1+ dissection; those with clinical T2–4 or N+ underwent D2 or D2+ dissection. Laparoscopic surgery was usually performed in patients with early stage gastric cancer, with an open procedure being the main surgical type used to treat advanced cancer. Patients who underwent total gastrectomy were given a Roux-en-Y reconstruction, while those undergoing distal sub-total gastrectomy underwent Roux-en-Y, Billroth I or Billroth II reconstructions. Proximal sub-total gastrectomy was carried out for proximal early stage gastric cancer, with esophagogastrostomy reconstructions being performed in these patients. Combined multi-organ resection was performed in patients with a locally advanced tumor suspected of invading adjacent organs for the purpose of R0 resection or simultaneous resection of other organs because of benign disease, such as cholecystectomy in patients with gallstones. A 6-mm silicon drain tube was placed in the sub-hepatic space and Morrison pouch, and a second drain positioned in the splenic fossa in those patients who underwent radical total gastrectomy or combined splenectomy and distal pancreatectomy; these drains were usually removed within 3 days after the initiation of feeding. A prophylactic antibiotic of a second-generation cephalosporin was administered intravenously to all patients 30 min before the first skin incision, and an additional dose was given if the operation time was >3 hours. The action of prophylactic antibiotics lasts for 3 to 5 days after the operation. Hospital discharge was usually permitted if patients had no abnormal physical signs, no abnormal laboratory findings and had the ability to consume an oral meal that met their physiological needs.

### Data collection

A number of clinicopathological factors that could potentially influence the likelihood of readmission were analyzed, such as age, gender, American Society of Anesthesiologists (ASA) score, a history of abdominal operations, body mass index (BMI), comorbidities (diabetes mellitus, hypertension, chronic pulmonary/kidney/liver disease, cardiovascular and cerebrovascular disease), pre-operative albumin and hemoglobin levels, type of gastrectomy, combined multi-organ resection, operation time, estimated intra-operative blood loss, peri-operative blood transfusion, post-operative hospital stays after the initial admission and the pathological TNM stage. Post-operative complications were defined as complications during the initial hospitalization and the severity was graded using a modified Clavien-Dindo classification^16^. As reported by Ahmad *et al*.^17^, grade I post-operative complications were excluded from our analysis since they had little clinical relevance and no detectable effect on the readmission rate. Major complications were defined as grade III or greater in accordance with the Clavien-Dindo classification.

### Definition of readmission

We used a 30-day readmission value defined as patients admitted to hospital within ≤30 days of discharge after the initial hospitalization. A time period of 30 days was adopted because previous studies have generally used a 30-day readmission rate as the evaluation index, allowing comparison of our results with those from previous similar studies. The admission records were carefully reviewed in order to clarify readmission only related to surgery or gastric cancer. Patients readmitted because of their adjuvant treatment plan such as chemotherapy, or due to other diseases, were excluded from the unplanned readmission group. The most significant problem was cited as the reason for readmission of patients who had multiple adverse effects. In addition, the time of readmission, type of treatment and potential risk factors for readmission were carefully evaluated. The study was approved by the Affiliated Cancer Hospital of Xiangya School of Medicine ethics committee, and informed consent was obtained from all patients.

### Statistical analysis

Statistical analyses were performed with IBM SPSS Statistics for Windows (Ver. 19, NY: IBM Corporation). Continuous data are reported as the mean ± standard deviation (SD) and any potential differences between groups reported as independent-sample *t*-test values. Categorical variables are expressed as numbers and percentages, and any significant differences between groups were assessed using a χ^2^ or a Fisher exact test. Risk factors for readmission were subjected to univariate analyses using a χ^2^ test to assess the effect of each factor. Multivariate logistic regression analysis was carried out for factors with a *P*-value ≤ 0.1 after univariate analysis. *P*-values < 0.05 were considered to represent statistical significance.

### Ethical approval

All procedures performed in studies involving human participants were in accordance with the ethical standards of the institutional and/or national research committee and with the 1964 Helsinki declaration and its later amendments or comparable ethical standards.

## Results

### Patient clinicopathological and operative outcomes

The baseline clinicopathological characteristics of the cohort of 2,023 patients are shown in Table 1. The median age of patients was 55.3 years (range, 19–83), and male patients accounted for the majority (66.1%). Total gastrectomy was performed in 420 patients (20.8%), sub-distal gastrectomy in 1,545 patients (76.4%), and sub-proximal gastrectomy in 56 patients (2.8%). Guided by the 7th edition of the UICC TNM classification, there were 522 (25.8%) stage I, 429 (21.2%) stage II, 1,060 (52.4%) stage III, and 12 (0.6%) stage IV patients. Laparoscopic or laparoscopy-assisted procedures were performed in 293 patients (14.5%). D2 or D2+ lymph node dissection was carried out in the majority of patients (n = 1,907, 94.3%) patients, with 181 patients (8.9%) having combined multi-organ resection. Cholecystectomy (n = 47, 2.3%) was the most common and most cases were performed because of cholecystitis or the presence of stones. Other organs such as the pancreas (n = 45), spleen (n = 39), colon (n = 23) and liver (n = 23) were mostly resected in order to achieve R0 resection. More than 1 organ simultaneous resection was necessary in 38 cases (21.0%) and the remaining 143 patients underwent only 1 organ resection. The mean operation time was 201 ± 53.7 min, and the mean estimated intra-operative blood loss was 205 ± 121 mL. The mean length of post-operative stays of the index hospitalization was 11.9 ± 6.5 days.

**Table 1:** Clinicopathological characteristics of patients based on 30-day readmission or not (n = 2,023).

Variables	Readmission group (n = 60)	Non-readmission group (n = 1,963)	χ^2^ or *t* value	*P* value
Sex				
Male	42 (70.0%)	1295 (66.0%)	0.42	0.52
Female	18 (30.0%)	668 (34.0%)		
Age (years)				
Median	56.80 ± 10.79	55.30 ± 10.78	1.06	0.29
Range	26–83	19–83		
Body Mass Index (kg/m^2^)	21.63 ± 3.00	21.80 ± 2.97	0.43	0.67
ASA score			2.26	0.52
1	10 (16.7%)	294 (15.0%)		
2	41 (68.3%)	1440 (73.3%)		
3	8 (13.3%)	220 (11.2%)		
4	1 (1.7%)	9 (0.5%)		
Smoking history	23 (38.3%)	846 (43.1%)	0.54	0.46
Any comorbidities	21 (35.0%)	589 (30.0%)	0.69	0.41
History of abdominal surgery	4 (6.7%)	201 (10.2%)	0.82	0.37
Neoadjuvant chemotherapy	5 (8.3%)	116 (5.9%)	0.61	0.44
Preoperative albumin (g/L)	38.83 ± 4.36	38.15 ± 4.58	1.13	0.26
Preoperative hemoglobin (g/L)	117.17 ± 27.00	118.69 ± 26.11	0.47	0.64
Complication due to the tumor^#^	16 (26.7%)	433 (22.1%)	0.72	0.40
Operation method			0.24	0.63
Open	50 (83.3%)	1680 (85.6%)		
Laparoscopy	10 (16.7%)	283 (14.4%)		
Type of resection			0.25	0.62
Subtotal gastrectomy	46 (76.7%)	1557 (79.3%)		
Total gastrectomy	14 (23.3%)	406 (20.1%) (20.7%)		
Extent of lymph node dissection			2.08	0.15
≥D2	54 (90.0%)	1853 (94.4%)		
<D2	6 (10.0%)	110 (5.6%)		
Reconstruction			5.35	0.15
B- I	28 (46.7%)	1203 (61.3%)		
B- II	9 (15.0%)	223 (11.4%)		
R - Y	21 (35.0%)	481 (24.5%)		
Esophagogastrostomy	2 (3.3%)	56 (2.9%)		
Combined multi-organ resection			4.52	0.03
Yes	10 (16.7%)	171 (8.7%)		
No	50 (83.3%)	1792 (91.3%)		
Tumor size (cm)	4.47 ± 2.49	4.04 ± 2.05	1.59	0.11
Tumor location			5.60	0.13
Upper	7 (11.7%)	164 (8.4%)		
Middle	9 (15.0%)	410 (20.9%)		
Lower	39 (65.0%)	1321 (67.3%)		
Diffuse	5 (8.3%)	68 (3.5%)		
Depth of invasion^a^			10.05	0.02
T1	9 (15.0%)	383 (19.5%)		
T2	5 (8.3%)	301 (15.3%)		
T3	9 (15.0%)	118 (6.0%)		
T4	37 (61.7%)	1161 (59.1%)		
Lymph node metastasis^a^			1.19	0.76
N0	20 (33.3%)	771 (39.3%)		
N1	10 (16.7%)	323 (16.5%)		
N2	12 (20.0%)	384 (19.6%)		
N3	18 (30.0%)	485 (24.7%)		
pTNM stage^a^			3.62	0.31
I	12 (20.0%)	510 (26.0%)		
II	10 (16.7%)	419 (21.3%)		
III	37 (61.7%)	1023 (52.1%)		
IV	1 (1.7%)	11 (0.6%)		
Intraoperative blood loss (mL)	227.8 ± 108.3	204.8 ± 121.2	1.46	0.15
Operation time (min)	206.50 ± 51.1	200.7 ± 53.8	0.83	0.41
Perioperative blood transfusion	16 (26.7%)	408 (20.8%)	1.22	0.27
Post-operative complications^b,c^			60.06	<0.001
None	40 (66.7%)	1802 (91.8%)		
Grade II	10 (16.7%)	117 (6.0%)		
Grade III or greater	10 (16.7%)	44 (2.2%)		
Initial hospital stays (days)	18.30 ± 11.16	11.66 ± 6.17	7.95	<0.001

Data are presented as mean ± SD or n (%).

ASA, American Society of Anesthesiology; B-1, Billroth I reconstruction; B- II, Billroth II reconstruction; R-Y, Roux-en-Y reconstruction.

^#^Including pyloric obstruction or bleeding.

^a^Tumor stages are based on 7th edition of the Union for International Cancer Control TNM classification.

^b^Defined as complications during the initial hospitalization.

^c^Based on the Clavien-Dindo severity classification of surgical complications.

Two hundred and eleven post-operative complications occurred in 181 patients of the entire cohort (8.9%), including 136 (64.5%) local and 75 (35.5%) systemic complications (Table 2). Of the local complications, intra-abdominal infection (n = 48) was the most common, followed by anastomotic leakage (n = 18), intestinal obstruction (n = 14), ascites (n = 11) and intra-abdominal bleeding (n = 10). Pulmonary infection (n = 51) and pleural effusion (n = 11) were the most common systemic complications. Following the Clavien-Dindo classification system, the incidence of stage II, IIIa, IIIb, IVa and IVb complications were 6.3% (n = 127), 0.7% (n = 15), 1.2% (n = 25), 0.6% (n = 12) and 0.1% (n = 2), respectively. Major complications, which were defined as grade III or greater based on Clavien-Dindo classification, occurred in 54 patients (2.7%).

**Table 2:** Post-operative complications that occurred prior to discharge (n = 211).

Complications	Number (%)
Local	136 (64.5%)
Intra-abdominal infection	48 (22.7%)
Anastomotic leakage	18 (8.5%)
Intestinal obstruction	14 (6.6%)
Intra-abdominal fluid collection	11 (5.2%)
Intra-abdominal bleeding	10 (4.7%)
Wound dehiscence	8 (3.8%)
Lymphatic fistula	7 (3.3%)
Gastrointestinal bleeding	7 (3.3%)
Pancreatic fistula	4 (1.9%)
Duodenal stump fistula	3 (1.4%)
Delayed gastric emptying	2 (0.9%)
Anastomotic stricture	2 (0.9%)
Systemic	75 (35.5%)
Pulmonary infection	51 (24.2%)
Pleural effusion	11 (5.2%)
Liver failure	2 (0.9%)
Urinary tract infection	2 (0.9%)
Cerebral infarction	2 (0.9%)
Pneumothorax	2 (0.9%)
Cardiac arrest	2 (0.9%)
Diabetic ketoacidosis	1 (0.5%)
Urinary retention	1 (0.5%)
Renal failure	1 (0.5%)

Sixty patients (3.0%) were readmitted to hospital within 30 days after the initial discharge. It was clear that patients who were readmitted had higher rates of deeper tumor invasion (T3–4), combined multi-organ resection and post-operative complications (Table 1, all *P* < 0.05). The mean initial post-operative hospital duration stay was 18.3 days for patients readmitted and 11.7 days for those not requiring readmission, a difference that was statistically significant (*P* < 0.001).

### Detailed clinical courses of the 60 patients readmitted

**Figure 1:**
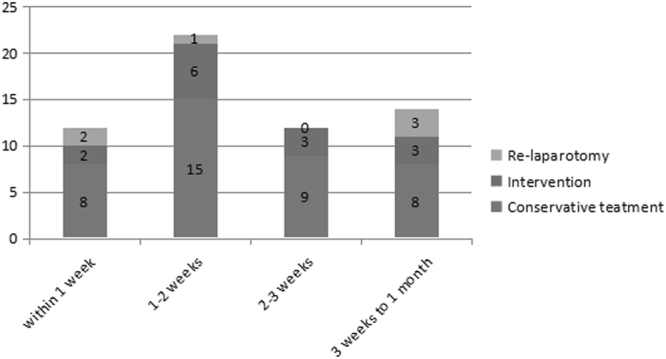
Type of treatment for 60 readmissions included 40 conservative treatments, 14 endoscopic or radiologic interventions, and 6 re-laparotomies.

Of the 60 patients who were readmitted, 42 (70.0%) were male and 18 (30.0%) female, with a mean age of 56.8 years (range, 26–83). Three patients were readmitted twice and 1 patient 3 times during the 30-day period. The median time between the index discharge and readmission was 14 days (range, 1–30). The time of, and the reasons for, readmission are shown in Table 3. The second week after the initial discharge was the most common time for readmission. Although the reasons for readmission varied widely, the vast majority of patients were readmitted due to local complications (n = 56, 90.0%), including intestinal obstruction (n = 10, 16.7%), intra-abdominal fluid collection (n = 9, 15.0%), abdominal pain (n = 7, 11.7%), nutritional difficulty (n = 4, 6.7%) and anastomotic leakage (n = 4, 6.7%). The type of treatment for the 60 readmitted patients included 6 repeat laparotomies, 14 endoscopic or radiologic interventions and 40 conservative therapies (Fig. 1). In those patients who underwent re-laparotomy, there were 3 intestinal obstructions, 1 intra-abdominal infection, 1 anastomotic leakage and 1 wound dehiscence. Five patients (8.3%) in the readmission group needed intensive care because of sudden cardiac arrest, shock, sepsis or respiratory failure. Moreover, 4 patients (6.7%) died from sudden cardiac arrest, gastrointestinal bleeding, sepsis or multiple organ dysfunctions. The median duration of hospitalization for the 60 patients who were readmitted was 8 days (range, 2–68).

**Table 3:** The reasons for admission and the timing of readmission after primary discharge (n = 60).

Reason for readmission	Within 1 week (n = 12)	1–2 weeks (n = 22)	2–3 weeks (n = 12)	3 weeks to 1 month (n = 14)
Local	11	21	11	13
Intestinal obstruction	3	2	3	2
Intra-abdominal fluid collection	1	5	0	3
Abdominal pain	1	3	2	1
Nutritional difficulty	0	3	1	0
Anastomotic leakage	1	3	0	0
Tumor recurrence	0	0	1	3
Nonspecific vomiting	0	2	1	1
Gastrointestinal bleeding	2	0	1	0
Intra-abdominal abscess	2	0	0	1
Anastomotic stricture	0	0	2	0
Delayed gastric emptying	0	2	0	0
Reflux esophagitis	1	0	0	1
Wound dehiscence	0	1	0	0
Wound infection	0	0	0	1
Systemic	1	1	1	1
Pneumonia	0	1	0	1
Anemia	0	0	1	0
Liver dysfunction	1	0	0	0

### Risk factors

On univariate analysis (Table 4), combined multi-organ resection, intra-operative blood loss ≥300 mL and post-operative complications were clarified as predictors for readmission. In addition, the invasion depth of T3–4 and the pTNM stage of III-IV appeared to be more common in the readmission group, though the differences were not statistically significant (*P* = 0.07 and 0.10, respectively). After multivariate analysis, including factors that had *P*-values ≤ 0.1 established by univariate analysis, post-operative complication (Odds Ratio [OR] = 5.116, 95% confidence interval [CI]: 2.885–9.073, *P* < 0.001) was shown to be an independent risk factor for readmission. Patients with more operative blood loss (≥300 mL) appeared to exhibit a trend towards a higher incidence of readmission (OR = 1.679, 95% CI: 0.948–2.974), but the difference was not statistically significant (*P* = 0.076) (Table 5). No other factors, including advanced age (≥70 years), BMI ≥ 25 kg/m^2^, comorbidities or total gastrectomy were identified as significant risk factors.

**Table 4:** Univariate analysis of possible predictors of risk for 30-day readmission following gastrectomy for gastric cancer (n = 2,023).

Variables	Readmission group (n = 60)	Non-readmission group (n = 1,963)	χ^2^ value	*P* value
Sex (Male: Female)	42:18	1295:668	0.42	0.52
Age(years) ≥70/<70	5:55	149:1814	0.05	0.83
BMI (kg/m^2^)≥25/<25	9:51	276:1687	0.04	0.84
ASA score ≥3/<3	9:51	229:1734	0.62	0.43
Comorbidity; yes/no	21:39	589:1374	0.69	0.41
Smoking history; yes/no	23:37	846:1117	0.54	0.46
History of abdominal surgery; yes/no	4:56	201:1762	0.82	0.37
Neoadjuvant chemotherapy; yes/no	5:55	116:1847	0.61	0.44
Preoperative albumin (g/L) <35/≥35	11:49	443:1520	0.60	0.44
Preoperative hemoglobin (g/L) <100/≥100	15:45	412:1551	0.56	0.45
Complication due to the tumor^#^; yes/no	16:44	433:14530	0.72	0.40
Operation method: open/laparoscopy	10:50	283:1680	0.24	0.63
Extent of gastric resection: subtotal/total	46:14	1557:406	0.25	0.62
Combined multi-organ resection; yes/no	10:50	171:1792	4.52	0.03
Intraoperative blood loss (mL): ≥300/<300	20:40	400:1563	5.94	0.02
Operation time (min): ≥240/<240	11:49	443:1520	0.60	0.44
Tumor size (cm); ≥5/<5	22:38	692:1271	0.05	0.82
Depth of invasion; T3–4/T1–2	46:14	1279:684	3.41	0.07
Lymph node metastasis; positive/negative	20:40	771: 1192	0.86	0.35
pTNM stage: III-IV/I-II	38:22	1034:929	2.66	0.10
Post-operative complications; yes: no	20:40	40:1802	45.14	<0.001
Perioperative blood transfusion; yes/no	16:44	408:1555	1.22	0.27

BMI body mass index, ASA American Society of Anesthesiologist.

^#^Including pyloric obstruction or bleeding.

**Table 5:** Multivariate analysis of possible predictors of risk for 30-day readmission following gastrectomy for gastric cancer (n = 2,023).

Variables	Odds Ratio [OR]	95% Confidence Interval [CI]	*P* value
Post-operative complications	5.116	2.885–9.073	<0.001
Operative blood loss ≥300 mL	1.679	0.948–2.974	0.076
Invasion depth of T3–4	1.481	0.654–3.352	0.347
Combined multi-organ resection	1.304	0.618–2.749	0.486
pTNM stage of III-IV	1.032	0.499–2.134	0.932

To further analysis which kinds of post-operative complications have the highest risk of readmission, the 181 patients who experienced post-operative complications were classified into subgroups according to the types of complications. As a result, patients who developed major complications (grade III or greater according to Clavien-Dindo classification, 18.5%) were confirmed to have the highest risk of requiring admission compared with those with minor (grade II according to Clavien-Dindo classification) or none complications (7.9% and 2.2%, *P* < 0.001). Similarly, patients who experienced local complications (14.0%) seemed more common to be readmitted than those with systemic or none complications (3.8% and 2.2%, *P* < 0.001).

## Discussion

In this retrospective study of a large cohort of patients from a single center specialized for cancer treatment in China, we found that need for readmission was mainly to manage severe complications during the early post-discharge period after radical gastrectomy for gastric cancer, such as adhesive intestinal obstruction and intra-abdominal infections. Because of its significant clinical and economic burden to the patient and medical system, reducing post-discharge complications and readmission has become a main goal for improving the quality of patient care and saving healthcare costs. Procedure-specific analysis of the incidence of, reasons, and risk factors for readmission is essential to develop targeted interventions to reduce readmission rates^1,5,11^. Several studies on readmission in patients who received gastrectomy surgery for gastric cancer have been reported to date, but the results differed significantly from the Eastern to the Western world. The majority of the research papers were published in the West, with the 30-day readmission rates ranging from 14.2–15.4%^2,12,17^. In Japan and South Korea, the 30-day rate readmission after gastrectomy was reported to be 2.2–7.5%^3,9,11,18^, where the patients generally presented with early stage gastric cancer and D1 or D1+ lymph node dissections were performed by a laparoscopic procedure. Unfortunately, the majority of patients with gastric cancer in China were diagnosed at an advanced stage. As shown in the present study, D2 lymphadenectomy using an open procedure is recommended by guidelines from the East^15^. The length of post-operative stay is usually longer in China, and the process of healthcare during hospitalization and post-discharge may vary widely compared to the West, Japan and South Korea; thus, it seems inappropriate to copy their experiences verbatim. Zhuang and colleagues (2015) published the only report in China that has analyzed in detail the 30-day readmission rate following gastrectomy for gastric cancer^19^. In their prospective study of 376 patients, they established that the incidence of readmission was 7.2% following gastrectomy. Given its relatively low incidence and the variety of reasons for readmission, this study was limited by its small cohort size. The conclusion should be cited with detailed interpretations and it will be necessary to validate the findings in a large-scale cohort of patients. As far as we are aware, this is the first study that has identified the incidence, reasons for, and the risk factors associated with readmission following radical gastrectomy for gastric cancer in a large number of Chinese patients.

The incidence of readmission was 3.0% in the present study, which was comparable with the 2.2–7.5% reported in Eastern hospitals^3,9,11,18^, but significantly lower than the 14.2–15.4% reported in Western hospitals^2,12,17^. Possible explanations include that the patients from the West were usually heavier, underwent gastrectomy with longer operative times, and had shorter hospital stays but with higher reported rates of post-operative complications. As reported by Schneider *et al*.^4^, an overwhelming majority of patients were readmitted to hospital within 14 days of discharge. In the present study 12 (20.0%) of the 60 readmissions occurred within 1 week and 22 (36.7%) between 1 and 2 weeks after discharge. Patients who experienced post-operative complications had a significant higher risk of readmission than those who did not, especially within the first 2 weeks following the initial discharge. Therefore, relatively closer follow-ups should be carried out for these patients than the usual schedule. If post-discharge complications were detected early and given facilitate intervention in the outpatient setting, some readmissions may have be avoided, as demonstrated by Antonoff *et al*. after lung resections and Hornick *et al*. after vascular surgery^20,21^.

As summarized in Table 3, the overwhelming majority of patients (90.0%) were readmitted due to local complications, including intestinal obstruction (n = 10), intra-abdominal fluid collection (n = 9), abdominal pain (n = 7), nutritional difficulty (n = 4) and anastomotic leakage (n = 4); while only 4 patients were readmitted because of systemic complications, such as pneumonia and liver dysfunction. Intestinal obstruction due to adhesion formation was the most frequent reason for readmission in the present study. Regardless of the type of abdominal surgery, adhesions are the most common cause of long-term complications, with the most severe consequence of adhesions being small bowel obstruction, which normally required readmission and further surgery^22^. It has been unequivocally demonstrated that the use of laparoscopy surgery reduces the incidence and severity of adhesions after colorectal and gastric cancer resection^9,23^. The proportion of readmissions due to local complications seemed relatively higher than in other similar studies^2,12^, but the findings are similar to those of Jeong and colleagues^11^ who clarified that 94.0% of patients were readmitted as a result of local complications. As the present study was based on data from a single institution specialized for cancer treatment, it is possible that patients who developed systemic post-discharge complications may have been readmitted into other general hospitals.

And among the 60 readmitted patients, 20 (33.3%) suffered from major complications. In detail, 14 (23.3%) patients and 6 patients (10.0%) required endoscopic or radiological interventions and re-laparotomy, respectively. Five (8.3%) patients requiring intensive care, and 4 (6.7%) patients died from sudden cardiac arrest or for other reasons. The rates of requiring intensive care and mortality in patients readmitted were significantly higher than those during the index hospitalization (2.3% and 0.3%, *P* = 0.003 and < 0.001, respectively). Of great importance is the fact that readmission may be related to major complications, requiring intervention or re-laparotomy and even result in death^24^. Thus, readmission indeed decreases the patients’ quality of life and becomes a significant clinical and economic burden to the patient and the medical healthcare system. On the other hand, the majority of the 60 readmitted patients (n = 40, 66.7%) needing only conservative treatments, some even requiring just observations because there were no substantive problems. Although it seems impossible to entirely prevent readmission, especially in the patients with higher risk. While given better pre-discharge management, and post-discharge patients counseling, some readmission of patients with lower risk may have be avoided. This assumption was echoed by Dawes AJ *et al*.^25^, who conducted a large-size retrospective study of readmissions for general surgery patients. They revealed that 21% of readmissions could be prevented and potential strategies were closer follow-ups, management in the outpatient setting and avoidance of premature discharge.

As reported in previous studies^4,12,13,17^, post-operative complication was identified as the only independent risk factor for readmission by multivariate analysis, a finding confirmed in the present study. Compared with those patients who experienced minor or none post-operative complications, patients who experienced a major complication were significantly more likely to be readmitted. This result was consistent with that reported by Ahmad *et al*.^17^, who argued that major complications (defined as grade II or higher according to Clavien-Dindo classification) was the most significant risk factor for predicting admissions. While some other authors have not similarly clarified the incidence of a post-operative complication as a risk factor for readmission, or included grade I morbidities in their analyses, which were believed to have little influence on the likelihood of readmission^3^. To the best of our knowledge, we first divided post-operative complications into local and systemic ones, and further analysis identified that patients who suffered local complications had a significant risk of readmission that those with systemic complications. One possible explanation was that systemic complications, such as pulmonary infections and pleural effusions, were relatively easy to manage than local complications, such as anastomotic leakage. And perhaps some other patients who developed systemic post-discharge complications may be readmitted into other general hospitals, considering the cancer-specialized nature of our institution.

Reddy *et al*.^26^ reported that about 80% of readmission cases after pancreatic procedures were associated with operative complications or infection. The question is raised whether reducing post-operative complications or prolonging post-operative hospital stay will lead to decreased readmission rates? There have been several ongoing studies exploring ways in which to reduce post-operative morbidity, but the related readmission rate has not been unequivocally shown to be decreased^12^. Surprisingly, the 30-day readmission rate increased with time. For example, patients discharged between 2001 and 2005 had a nearly 50% chance of being readmitted to hospital in comparison with those discharged between 1986 and 1990^4^. This finding may be because, for the majority of patients, readmissions were due to new post-discharge symptoms associated with surgery, but distinct from in-hospital post-operative morbidities^13^. In this study, only 11.1% (n = 20) of patients who developed post-operative complications were readmitted, while the majority (n = 40, 66.7%) was readmitted because of post-discharge symptoms or complications. Similarly, in a large-scale study that evaluated readmission rates in patients who underwent general surgery, Tevas and colleagues^27^ reported that only 7% of patients with pre-discharge complications were readmitted compared to more than half (56%) of those with post-discharge complications (*P* < 0.001). It seems that a direct relationship between post-operative complications and readmission only exists in a small proportion of patients, indicating that to decrease readmission rates, more attention should be paid to preventing post-discharge events, rather than focusing on decreasing the incidence of pre-discharge complications^12^. But it is still remains uncertain whether post-operative and post-discharged complications are directly related. On the other hand, althouth length of stay had an overall minimal impact on the risk of admission, early discharge did increase the risk of readmission among patients with a postoperative complication^4^. Thus in theory, appropriately prolonging the length of stay to manage the postoperative complication completely could reduce the incidence of readmission. But the standards for discharge of thses patients have not been well defined, and an prospective study is necessary in the future.

The present research had a number of limitations; one being that it was a single-center retrospective study meaning that the conclusions cannot be easily extended to other Chinese hospitals. Furthermore, whether the patient was readmitted was significantly affected by the patients’ preference and socio-economic conditions, but unfortunately there is no unified standard to measure these parameters. Finally, the conclusions of this study are based on data obtained from a single high-volume institution specialized to treat only cancer. Thus, possible readmission to other hospitals was not accounted for, especially those patients with systemic post-discharge complications, leading to a potential underestimation of the readmission rate. However, the prospectively registered high-volume sample database that collected and stored detailed data should minimize the impact of confounding factors and improve the credibility of the conclusions. Nevertheless, the present study identified in a large cohort of patients in China specific factors associated with the need for patients to be readmitted to hospital after radical gastrectomy for gastric cancer.

In summary, this retrospective study from a high-volume center specialized for the treatment of cancer in China has shown that the readmission rate following radical gastrectomy for gastric cancer is 3.0%. Post-operative complications are the only identified independent risk factor for readmission. Thus appropriate strategies on discharge and close follow-ups for these high-risk patients, especially those who suffered major or local post-operative complications, should be drawn up in order to enhance significantly their quality of care.
